# The Role of Visual Electrophysiology in Systemic Hereditary Syndromes

**DOI:** 10.3390/ijms26030957

**Published:** 2025-01-23

**Authors:** Minzhong Yu, Emile R. Vieta-Ferrer, Anas Bakdalieh, Travis Tsai

**Affiliations:** 1 Department of Ophthalmology and Visual Sciences, University Hospitals Eye Institute, Case Western Reserve University, Cleveland, OH 44106, USA; 2 Cole Eye Institute, Cleveland Clinic Foundation, Cleveland, OH 44106, USA; 3 Department of Ophthalmology, Cleveland Clinic Lerner College of Medicine of Case Western Reserve University, Cleveland, OH 44195, USA; 4 Jules Stein Eye Institute, University of California, Los Angeles, CA 90095, USA; evieta@mednet.ucla.edu; 5 College of Medicine, Northeast Ohio Medical University, Rootstown, OH 44272, USA; abakdalieh@neomed.edu; 6 School of Medicine, Case Western Reserve University, Cleveland, OH 44106, USA; tdt37@case.edu

**Keywords:** full-field electroretinography, multifocal electroretinography, pattern electroretinography, visual evoked potentials, electrooculography, hearing loss syndromes, mitochondrial disorders, obesity syndromes, retinal degeneration

## Abstract

Visual electrophysiology is a valuable tool for evaluating the visual system in various systemic syndromes. This review highlights its clinical application in a selection of syndromes associated with hearing loss, mitochondrial dysfunction, obesity, and other multisystem disorders. Techniques such as full-field electroretinography (ffERG), multifocal electroretinography (mfERG), pattern electroretinography (PERG), visual evoked potentials (VEP), and electrooculography (EOG) offer insights into retinal and optic nerve function, often detecting abnormalities before clinical symptoms manifest. In hearing loss syndromes like Refsum disease, Usher syndrome (USH), and Wolfram syndrome (WS), electrophysiology facilitates the detection of early retinal changes that precede the onset of visual symptoms. For mitochondrial disorders such as maternally-inherited diabetes and deafness (MIDD), Kearns–Sayre syndrome (KSS), and neuropathy, ataxia, and retinitis pigmentosa (NARP) syndrome, these tests can be useful in characterizing retinal degeneration and optic neuropathy. In obesity syndromes, including Bardet-Biedl syndrome (BBS), Alström syndrome, and Cohen syndrome, progressive retinal degeneration is a hallmark feature. Electrophysiological techniques aid in pinpointing retinal dysfunction and tracking disease progression. Other syndromes, such as Alagille syndrome (AGS), abetalipoproteinemia (ABL), Cockayne syndrome (CS), Joubert syndrome (JS), mucopolysaccharidosis (MPS), Neuronal ceroid lipofuscinoses (NCLs), and Senior–Løken syndrome (SLS), exhibit significant ocular involvement that can be evaluated using these methods. This review underscores the role of visual electrophysiology in diagnosing and monitoring visual system abnormalities across a range of syndromes, potentially offering valuable insights for early diagnosis, monitoring of progression, and management.

## 1. Introduction

Visual electrophysiology is a valuable tool for the diagnosis and monitoring the progression of ocular manifestations in various systemic syndromes. This review examines its clinical application in a selection of hereditary syndromes, including some associated with hearing loss, mitochondrial dysfunction, obesity, and other systemic conditions. By providing objective measurements of visual pathway function, electrophysiological techniques such as electroretinography (ERG), multifocal electroretinography (mfERG), visual evoked potentials (VEP), and electrooculography (EOG) have become integral to understanding the retinal and visual system involvement in these complex disorders.

Electrophysiology can facilitate the early identification of retinal changes, often appearing before overt clinical symptoms, in syndromes associated with hearing loss, such as Refsum disease, Usher syndrome, and Wolfram syndrome. Similarly, in mitochondrial diseases like maternally-inherited diabetes and deafness (MIDD), Kearns–Sayre syndrome, and neuropathy, ataxia, and retinitis pigmentosa (NARP) syndrome, retinal and optic nerve dysfunctions are common, making electrophysiological assessments useful for early diagnosis and effective disease management. Obesity-related syndromes, including Alström syndrome, Bardet–Biedl syndrome, and Cohen syndrome, commonly exhibit progressive retinal degeneration as a key feature. Visual electrophysiology can be used to track the onset and progression of retinal dysfunction, offering valuable insights into disease mechanisms and potential therapeutic responses. Other multisystem syndromes, such as abetalipoproteinemia (Bassen–Kornzweig syndrome), Alagille syndrome, Cockayne syndrome, Joubert syndrome, mucopolysaccharidosis, neuronal ceroid lipofuscinoses (Batten’s disease), and Senior–Løken syndrome, also present ocular complications that can be evaluated using these techniques. Electrophysiology can, at times, detect functional impairments even in the absence of apparent structural damage, offering crucial insights into the early and often subclinical stages of these disorders.

Full-field electroretinography (ffERG) is one of the most commonly used electrophysiological tests for assessing overall retinal function [1]. It measures the electrical responses of the entire retina to light stimuli, evaluating both rod and cone photoreceptor activity. This technique provides a global view of retinal function and is particularly useful in detecting diffuse retinal disorders commonly associated with hereditary etiologies.

Multifocal electroretinography (mfERG) complements ffERG by providing localized data on retinal function across the central visual field. It measures multiple ERG responses simultaneously from different retinal regions, allowing for a detailed mapping of retinal dysfunction [2]. This technique is especially beneficial in detecting localized retinal abnormalities, such as those found in Usher syndrome and Alström syndrome, where central vision is impacted at an early age.

Pattern electroretinography (PERG) is a specialized electrophysiological test that evaluates the function of the retinal ganglion cells (RGCs) and their axons in response to visual stimuli [3]. PERG is elicited using contrast-reversing stimuli, such as black-and-white checkerboards or gratings, without significant changes in overall luminance. This test is particularly sensitive to conditions that affect RGCs, such as glaucoma, optic neuropathies, and certain neurodegenerative diseases. The PERG waveform typically consists of an initial positive deflection (P50) and a subsequent negative deflection (N95), which are closely associated with photoreceptor-driven and ganglion cell-driven responses, respectively.

Visual evoked potential (VEP) evaluates the functional integrity of the optic nerve and visual pathways by measuring the brain’s electrical response to visual stimuli [4]. VEP is useful for evaluating post-retinal visual pathway disturbances, such as optic neuropathies that often occur in mitochondrial diseases like Kearns–Sayre syndrome and NARP syndrome. It is also useful for detecting subclinical optic nerve dysfunction in syndromes with ocular involvement. Pattern VEP (PVEP) that is elicited by pattern reversal or on–off stimulation is mainly used for the patients who can still see the pattern stimulation. For the subjects who cannot see the pattern stimulation, flash VEP (FVEP) can be used, although the variation of the FVEP components is much larger than that of PVEP.

Electrooculography (EOG) is another important tool used to assess the function of the retinal pigment epithelium (RPE), a cell layer essential for photoreceptor health. EOG measures the standing potential generated between the cornea and the retina, particularly in response to light and dark adaptation cycles [5]. Disorders affecting the RPE, such as Bassen–Kornzweig syndrome and certain forms of neuronal ceroid lipofuscinoses, can be detected early using EOG, even when the retinal structure appears normal on imaging. A summary of the electrophysiological tests and their characteristics are highlighted in Table 1.

Additional imaging exams are also valuable in diagnosis. A multimodal diagnostic approach provides multifaceted data that helps clinicians shorten the list of potential diagnoses. Each diagnostic modality offers unique insights, and their integration allows for cross-validation of findings, reducing ambiguity and refining the diagnostic possibilities. By providing structural, functional, and biochemical data, multimodal exams prioritize the most likely diagnoses and exclude less probable ones, streamlining the diagnostic process and improving accuracy. This approach enhances efficiency, reduces reliance on single tests, and ensures a more confident and precise diagnosis, especially for complex or multi-system diseases.

This review aims to summarize the current applications of visual electrophysiology across these diverse syndromes, highlighting its role in early diagnosis, monitoring disease progression, and advancing the understanding of underlying pathophysiological mechanisms.

## 2. Hearing Loss Syndromes

### 2.1. Refsum Disease

Refsum disease, also known as heredopathia atactica polyneuritiformis or phytanic acid storage disease, is an autosomal recessive metabolic disorder, a condition characterized by disruptions in normal biochemical processes, caused by peroxisomal dysfunction, leading to the accumulation of phytanic acid (a branched very long-chain fatty acid). Refsum disease presents in two primary forms: adult-onset and infantile-onset. Its primary features include pigmentary retinal degeneration (rod–cone RP type), progressive sensorineural hearing loss, cerebellar ataxia, and peripheral polyneuropathy.

The adult-onset form of Refsum disease results from mutations in at least two genes (*PHYH* and *PEX7*), whereas the infantile-onset form is associated with mutations in two other genes (*PEX1* and *PEX2*). These genes are essential for peroxisomal function, and their dysfunction is the underlying etiology of all forms of Refsum disease [6]. Peroxisomal dysfunction disrupts the α-oxidation pathway responsible for degrading phytanic acid, leading to its accumulation in tissues and in the bloodstream. Elevated phytanic acid levels are toxic to retinal cells, particularly rod photoreceptors, inducing cellular stress and degeneration. As rod cells deteriorate, cone photoreceptors undergo secondary degeneration, ultimately resulting in the rod–cone retinopathy characteristic of Refsum disease, closely resembling retinitis pigmentosa [7]. The rod photoreceptors and cone photoreceptors can be distinguished by their location and function. Rod cells are predominantly distributed in the peripheral retina, where they enable scotopic vision in low-light conditions, while cone cells are concentrated in the central retina, particularly in the fovea, supporting photopic vision and color perception.

The retinal degeneration in Refsum disease manifests as a rod–cone, RP-like retinopathy, with a significant reduction or complete absence of ERG rod and cone responses, which is similar to those of non-syndromic RP. The differential diagnosis for Refsum disease includes Usher and pseudo-Usher syndromes, as well as mitochondrial conditions like neuropathy, ataxia, and retinitis pigmentosa (NARP) syndrome and Kearns–Sayre syndrome, which also involve hearing loss. A diagnosis of Refsum disease can be made through genetic testing and serological testing positive for elevated phytanic acid levels.

Early recognition of Refsum disease is critical to mitigate its severity. Management of this condition includes a low-phytanic acid high calorie diet, avoidance of fasting, and plasmapheresis or lipid apheresis (in extreme cases). It is recommended that ERG should be used to monitor the effects of treatment on visual recovery [6,8].

### 2.2. Usher Syndrome

Usher syndrome (USH) is an autosomal recessive disorder characterized by hearing loss and vision impairment. It is the leading cause of deaf-blindness, with an incidence of 1–6 per 100,000 individuals. First described in 1914 by British ophthalmologist Charles Usher, the disorder is categorized into three primary types (I, II, and III) based on the severity and progression of symptoms [9]. Type I is characterized by profound congenital non-progressive hearing loss, often with vestibular dysfunction and visual symptoms appearing within the first two decades, frequently presenting severe retinitis pigmentosa (RP). Type II involves mild to moderate congenital hearing loss, predominantly at high frequencies, with varying degrees of vestibular dysfunction present at birth. Reportedly, 100% of USH Type II patients develop retinitis pigmentosa. However, the age of onset of RP is variable, typically starting in late adolescence or early adulthood, and some individuals may not exhibit symptoms until later in life, resulting in delayed diagnosis. Types I and II make up over 90% of USH cases. Type III, on the other hand, is associated with mild to moderate progressive hearing loss beginning after speech development, varying degrees of vestibular dysfunction, and RP symptoms that manifest within the first few decades, typically progressing more rapidly than type II [10].

In recent years, USH type IV has been recognized. This form is characterized by ring-shaped retinal atrophy extending temporally to the vascular arcades and nasally beyond the optic nerve while relatively sparing the mid- and far-periphery. Over time, pigment migrates within the atrophic areas, creating bone-spicule-like pigmentary changes and pigment clumps, eventually affecting the central macula. Visual symptoms for USH Type IV typically develop between the ages of 40 and 60. The patients have no significant vestibular system abnormalities and have moderate to severe sensorineural hearing loss starting at a relatively late age, usually after 40, but occasionally as early as childhood [11,12,13].

USH is primarily caused by pathogenic variants in genes responsible for the development and function of sensory cells in the cochlea of the inner ear and the retina. These variants disrupt the normal structure and function of these cells, leading to progressive degeneration of both hearing and vision. To date, variants in at least 13 genes have been implicated in Usher syndrome, with each gene linked to a specific subtype of the disorder. These genes play key roles in processes such as hair cell development in the inner ear, photoreceptor function in the retina, and the maintenance of sensory cell structures. Variants in at least six of these genes are associated with type I (most commonly *MYO7A* and *CDH23*) [14,15,16,17,18,19,20,21,22]. Up to 15% of USH Type I cases do not have a presently identifiable genetic etiology [23]. The *USH2A*, *ARGRV1*, *WHRN*, and *PDZD7* genes are associated with type II [24,25,26]. The *CLRN1* and *HARS1* genes are associated with type III [27]. Recently, certain variants of the arylsulfatase G (*ARSG*) gene have been linked to USH type IV [11,12,28,29].

ffERG is a useful diagnostic tool for Usher syndrome, as it assesses how these gene abnormalities impact retinal cells. The pathogenic variants linked to USH typically result in characteristic alterations in photoreceptor activity, which are detectable as reduced or absent rod and cone responses on ffERG. For example, individuals with *MYO7A* or *USH2A* mutations often display early attenuation of rod-driven scotopic responses, demonstrating the degeneration of peripheral photoreceptors. These functional deficits can be detected before structural changes become apparent on imaging, making ffERG an useful diagnostic modality for early disease detection and monitoring progression. By linking specific gene variants to distinct electrophysiological profiles, ffERG not only supports diagnostic accuracy but also provides insights into the pathophysiology underlying Usher syndrome [30].

**Figure 1:**
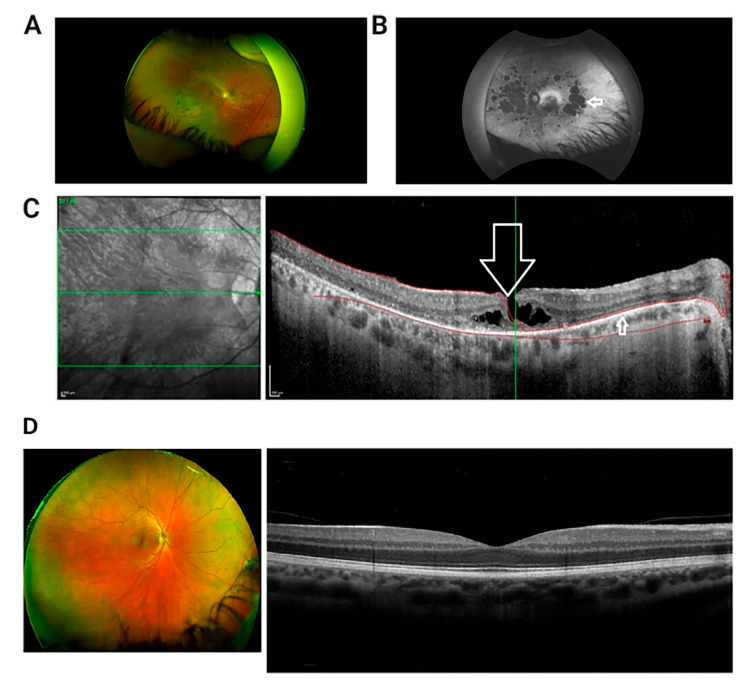
Multi-modal retinal imaging of a patient with USH Type IIA. (**A**) Wide field retinal photograph displaying mid peripheral bone spicule pigmentation and perivascular atrophy. (**B**) Red-free wide-field photograph showing widespread areas of chorioretinal atrophy, presented as hypofluorescent/dark patches (indicated by arrow). (**C**) OCT image of macula in right eye presenting lamellar macular hole (indicated by large arrow). There is disruption of the inner retinal layers, but the thinned retinal pigment epithelium is intact (smaller arrow). Green Lines: The central green line corresponds to the scan plane of the B-scan image shown on the right, providing a cross-sectional view of the retina at that specific depth. The upper and lower green lines demarcate the range of the retinal volume being scanned and analyzed. Red Curves: The red curves delineate the area of interest within the retina, highlighting the anatomical structures withpathological changes. (**D**) Wide field retinal photograph and OCT of a normal subject [32].

**Figure 2:**
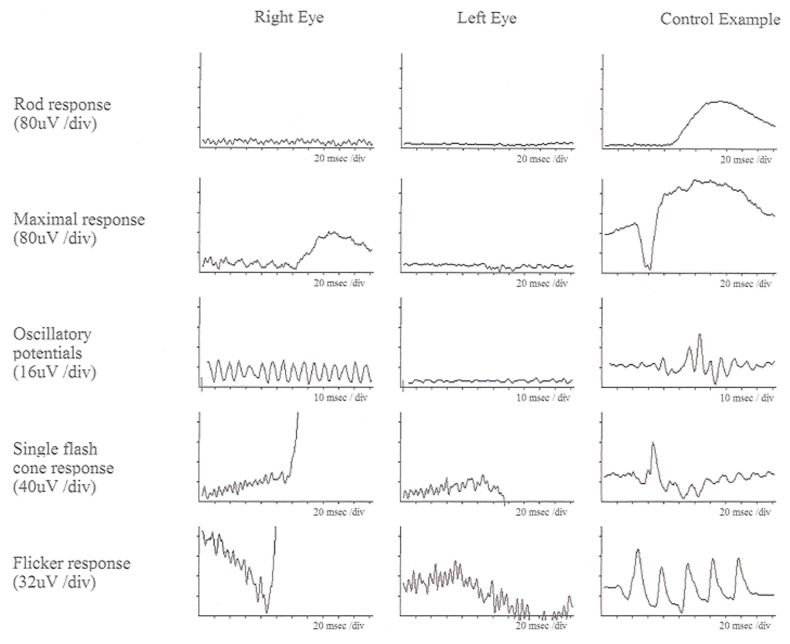
ffERG responses of a patient with USH Type IIA at age of 52. Left column (right eye), middle column (left eye), and right column (control data) are displayed. All rod and cone responses are severely reduced compared to control data [32].

**Figure 3:**
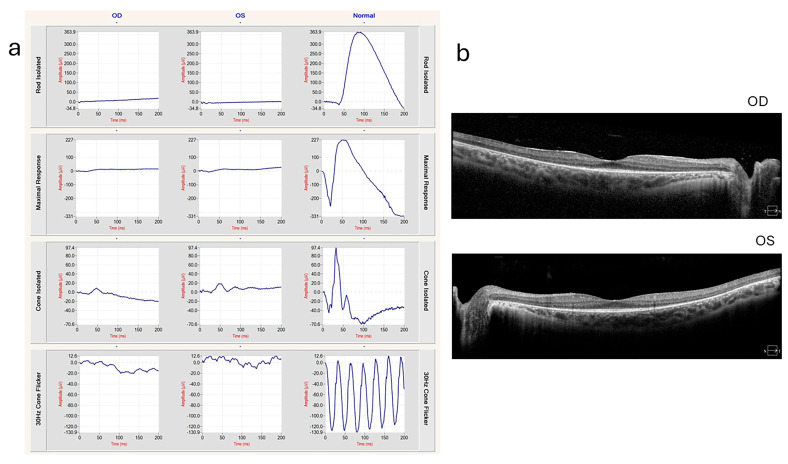
ffERG and OCT of a 20-year-old patient with Usher syndrome type II (biallelic pathogenic variants in *USH2A*: c.2276G>T, c.7595-2144A>G) (**a**) ERG showing extinguished rod responses and severely diminished cone responses. (**b**) OCT shows ellipsoid zone loss with foveal sparing, indicating photoreceptor degeneration. These images were provided by Michael B. Gorin MD, PhD, at the Jules Stein Eye Institute, University of California, Los Angeles, with the patient’s informed consent.

A diagnosis of USH ideally involves a thorough evaluation by a multidisciplinary team of healthcare professionals, including audiologists, ophthalmologists, geneticists, and other specialists. The key components of the diagnostic process are as follows: audiometric tests, including pure-tone audiometry, speech audiometry, and auditory brainstem response (ABR), assessing the degree and type of hearing loss; ophthalmologic evaluations, including dilated eye examination, visual field testing, ERG, and optical coherence tomography (OCT) [31], for the evaluation of retinal structure (Figure 1) and function (Figure 2 and Figure 3); and genetic testing to identify disease-causing variants in genes associated with Usher syndrome.

In individuals with USH, ffERG often detects abnormalities indicative of retinitis pigmentosa (RP), a hallmark of the condition. ffERG rod responses, which are responsible for vision in low-light conditions, typically exhibit significant abnormalities early in the disease, reflecting the early and progressive degeneration of rod photoreceptors. This degeneration leads to symptoms such as night blindness and peripheral vision loss. Cone responses also show abnormalities, though these changes may occur later and can progress more slowly compared to the changes in rods. The reduction or absence of both rod and cone responses on ffERG can help confirm the diagnosis of USH and differentiate it from other retinal disorders (Figure 2). In a 20-year-old patient with Usher syndrome type II (biallelic pathogenic variants in *USH2A:* c.2276G>T, c.7595-2144A>G), multimodal retinal assessment identified fundus changes. OCT imaging of both eyes (OD and OS) demonstrated symmetrical thinning of the outer retinal layers with disruption of the ellipsoid zone, a hallmark of photoreceptor degeneration frequently observed in *USH2A*-associated RP. Functionally, the ffERG showed a severe reduction of both rod and cone responses, reflecting advanced retinal dysfunction and consistent with the typical progression of *USH2A*-related disease, where rod impairment precedes and predominates over cone loss. Compared to the data in Figure 2, our data with biallelic pathogenic variants in *USH2A* shows similar level of thinning outer nuclear layer of retina in much earlier age (Figure 3). mfERGs in USH patients can show reduction of response densities that is more severe in the peripheral retina than in the central retina. Similar to retinitis pigmentosa, mfERG P1 implicit time in USH type II can be significantly delayed in the peripheral retina; it is not observed in USH type I [33]. The electrophysiological data can play a significant role in monitoring disease progression, aiding in classification, guiding clinical management, and evaluating the effectiveness of potential treatments.

### 2.3. Wolfram Syndrome

Wolfram syndrome (WS, also known as WFS1 Spectrum disorder) is a rare autosomal recessive disorder, also known by the acronym DIDMOAD, which stands for its main features: diabetes insipidus (DI), insulin-dependent diabetes mellitus (DM), progressive optic atrophy (OA), and deafness (D). It is characterized by symptoms including diabetes insipidus, insulin-dependent diabetes mellitus, optic atrophy, and deafness. In addition, these patients are at increased risk of early death from progressive brainstem atrophy and urinary tract atony.

WS can also present with a range of other clinical ocular manifestations, particularly affecting the posterior segment, which include: (1) Optic atrophy: One of the earliest and most consistent features of WS. This begins in childhood or early adolescence, leading to a reduction in visual acuity, color vision deficits, and peripheral vision loss. (2) Pigmentary retinopathy: This retinopathy resembles retinitis pigmentosa, with “bone spicule” intraretinal pigmentation and attenuation of retinal vessels. (3) Electrophysiological abnormalities: Visual electrophysiological tests, such as VEP and ffERG, are often abnormal in WS patients. VEP often reveals reduced amplitude or delayed implicit time proportional to the severity of optic atrophy [34,35]. ffERG abnormalities in rod and cone responses are observed, which is used to detect early retinal changes in WS. Studies have reported that patients with WS1 exhibit abnormalities in both rod and cone responses on ffERG, even before significant visual symptoms become apparent [36]. These electrophysiological alterations can precede observable structural changes in the retina, emphasizing the value of ERG for early detection and monitoring of retinal involvement in WS.

To date, only one gene, *WFS1,* has been associated with Wolfram syndrome Type I. *WFS1* codes for wolframin, a transmembrane glycoprotein involved in the regulation of endoplasmic reticulum (ER) stress and calcium homeostasis [37,38]. Its deficiency gives rise to cellular stress and apoptosis in retinal ganglion cells (RGCs), resulting in progressive optic atrophy [39]. Notably, the gene *CISD2* has been associated with Wolfram syndrome Type II (WS2). This gene encodes a small protein of the endoplasmic reticulum intermembrane (ERIS) [40,41]. A pathogenic *WFS2* variant disrupts calcium ion homeostasis and mitochondrial function, contributing to retinal cell degeneration. Although WFS2 has been reported to exhibit some findings similar to WFS1, the complete clinical spectrum of this disease has not been established due to the small number of cases. Confirmatory diagnosis of WSI or WSII involves genetic testing of the *WFS1* and *CISD2* genes. A summary of the characteristics of the hearing loss syndromes and their ocular manifestations are highlighted in Table 2.

## 3. Mitochondrial Diseases

### 3.1. Maternally-Inherited Diabetes and Deafness Syndrome

Maternally-inherited diabetes and deafness (MIDD) syndrome is a rare mitochondrial disorder that primarily affects the auditory and visual systems alongside insulin-dependent diabetes mellitus. Mitochondrial disorders are a group of diseases caused by dysfunctions in the mitochondria, the energy-producing organelles of cells, which can affect multiple organ systems, particularly those with high energy demands. The genetic basis of MIDD is often linked to the m.3243A>G variant in the mitochondrial DNA, specifically within the *MTTL1* gene [42]. These patients typically present with a spectrum of symptoms that can include bilateral macular pattern dystrophy (MPD), hearing loss, external ophthalmoplegia, ptosis, cardiomyopathy, myopathy, renal problems, and neuropsychiatric symptoms [42,43,44]. The type, extent, and severity of macular involvement vary between patients and within families [45,46,47]. The progression of the MIDD-MPD syndrome is relatively slow [48,49]. In its earlier stages, it can remain asymptomatic for years until functional loss around the fovea develops, causing reading difficulties. In more advanced stages, MIDD patients can develop macular atrophic areas resembling geographic atrophy and may progress to involve the fovea, which can lead to severe central vision loss. Notably, these diabetic patients hardly ever develop severe diabetic retinopathy [50]. In some cases, neovascular complications that resemble age-related macular degeneration (AMD) may develop, making it difficult to establish an accurate diagnosis. The triad of insulin-dependent diabetes mellitus, hearing loss, and macular dystrophy, however, is usually seen in patients in their 40’s to 50’s, an unusually early onset for AMD. Thus, regardless of whether it is associated with a maternal history of diabetes, this triad indicates a work-up for serum lactate and pyruvate levels (usually elevated in MIDD-MPD syndrome patients), as well as genetic testing.

From the visual electrophysiological standpoint, the mfERG usually detects abnormalities early-on in the disease with decreased response amplitudes, corresponding to the areas most affected by the retinal disease. Patients who retain normal visual acuity but exhibit abnormal mfERG findings have been identified, leading to closer monitoring and early intervention. In addition, many MIDD-MPD patients also display reduced PERG P50 amplitudes [51]. ffERG is generally not a sensitive diagnostic test in MIDD-MPD patients, although, in more advanced stages, patients can show abnormal rod and cone responses [52]. It is important to clarify that neither of these visual electrophysiological tests is diagnostic for the MIDD-MPD syndrome. In addition to the noted pathognomonic clinical triad, fluorescein angiography and fundus autofluorescence are useful from the ophthalmological standpoint. These tests are typically diagnostic for the linear spoke–wheel RPE macular changes characterizing the MPD of the MIDD-MPD syndromic association and are useful in confirming this diagnosis.

Integrating visual electrophysiology into the clinical management of MIDD provides several important implications. Firstly, these tests facilitate early detection of retinal abnormalities, often before patients experience significant visual symptoms. Early identification allows for timely intervention, potentially preserving vision and improving quality of life. Secondly, visual electrophysiology provides a non-invasive means to monitor disease progression. Regular testing can track changes in retinal function over time, allowing clinicians to adjust treatment plans as needed. This monitoring is particularly relevant in the context of emerging therapies, such as pro-mitochondrial dietary regimens (e.g., coenzyme Q-10) [45,53], which have reduced insulin requirements and may positively impact the macular phenotype.

### 3.2. Kearns–Sayre Syndrome

Kearns–Sayre syndrome (KSS) is a rare mitochondrial disorder caused by large deletions in mitochondrial DNA (mtDNA) [54], which are most often de novo variants, rather than inherited. Symptoms of KSS usually manifest before the age of 20, with the eyes being the first organs affected. KSS shares some clinical features with Usher syndrome (sensorineural hearing loss) and Refsum disease (cardiomyopathy, increased protein in the cerebrospinal fluid, sensory neuropathy). However, cone–rod type retinal degeneration (CORD), characterized by atrophy of the retinal pigment epithelium (RPE) at the posterior pole and around the optic disc, is a unique finding not seen in these other conditions. Patients with KSS often experience light sensitivity more often than night blindness, and their central vision is typically more impaired than their peripheral vision. Additional hallmark features include bilateral chronic progressive external ophthalmoplegia (CPEO) with ptosis, heart conduction abnormalities that increase the risk of sudden heart block, ragged red fibers (RRF) in the striated muscles observed during muscle biopsy, and lactic acidosis. To prevent severe cardiac complications, prophylactic pacemaker implantation is advised [55,56].

KSS may also present with other symptoms, such as hearing loss, diabetes mellitus, muscle weakness in the limbs, kidney issues, developmental delays, short stature linked to growth hormone deficiency, ataxia, seizure disorders, hypothyroidism, and delayed sexual maturation [57,58]. In rarer cases, corneal opacities may develop due to corneal endothelial dysfunction, sometimes necessitating a corneal transplant [59]. However, recent studies have shown that coenzyme Q-10 supplementation can improve corneal conditions [60,61] and might also positively impact CPEO and other disease manifestations. Genetic confirmation of KSS is typically achieved through mtDNA analysis on blood. However, falsely negative results can occur due to low heteroplasmy in that tissue. If the genetic test on blood is negative but suspicion remains high, genetic analysis of a muscle biopsy could confirm the diagnosis [62].

Visual electrophysiology is useful in the clinical management of KSS, providing valuable insights into the extent and nature of retinal dysfunction. In KSS, patients often exhibit abnormalities in ffERG rod and cone responses [63]. In its early stages, KSS can reveal subtle ERG changes in retinal function before clinical symptoms become evident.

### 3.3. Neuropathy, Ataxia, and Retinitis Pigmentosa Syndrome

Neuropathy, ataxia, and retinitis pigmentosa (NARP) syndrome is a rare mitochondrial disorder characterized by a combination of retinal and neurological manifestations. The condition arises due to pathogenic variants in the mitochondrial DNA *MTATP6* gene, which encodes subunit 6 of the mitochondrial H(+)-ATPase and plays a critical role in mitochondrial energy production [64,65,66]. Unlike Kearns–Sayre syndrome (KSS), NARP is usually inherited maternally, as expected with mitochondrial conditions. The likelihood of disease manifestation also depends on the degree of heteroplasmy (also known as mutant load). Notably, pathogenic variants in this gene have also been associated with Leigh syndrome and Leber’s Congenital Optic Neuropathy.

NARP syndrome typically manifests in childhood or early adulthood and presents with symptoms of night blindness, photophobia, and peripheral visual field loss, which are similar to retinitis pigmentosa (RP). However, unlike classic RP, NARP-related retinopathy is characterized by a “salt and pepper” appearance of the retina, with coarse retinal pigment epithelium (RPE) mottling rather than the diffuse bone spicule deposits seen in typical RP. The visual impairment often worsens due to the development of optic atrophy and ophthalmoplegia. Beyond its ocular manifestations, NARP syndrome is linked to a variety of systemic issues, including peripheral neuropathy, ataxia associated with corticospinal tract degeneration, hearing loss, short stature, proximal muscle weakness, learning difficulties, seizures, sleep apnea, and cardiac arrhythmias [67]. Progressive kidney dysfunction, potentially leading to renal failure and the need for dialysis, has also been reported [68]. Given the variability in symptom onset and severity, diagnosing NARP syndrome can be difficult, particularly when attempting to distinguish it from non-syndromic RP, Usher syndrome, and Refsum disease.

In NARP syndrome, visual electrophysiology helps in the early detection of retinal dysfunction, often before significant visual symptoms develop. ffERG is particularly useful in identifying the characteristic cone–rod pattern of degeneration, with both cone and rod responses often becoming non-recordable by the third decade of life [69]. A summary of the characteristics of the mitochondrial diseases and their ocular manifestations are highlighted in Table 3.

## 4. Obesity Syndromes

### 4.1. Bardet–Biedl Syndrome

First identified by Georges Bardet in 1920 [70] and Artur Biedl in 1922, Bardet–Biedl syndrome (BBS) is a rare autosomal recessive ciliopathy that occurs in approximately 1 in every 140,000 to 160,000 live births [71]. Individuals with BBS often suffer from progressive retinal degeneration, resulting in visual impairment and, in severe cases, blindness. This retinal degeneration typically presents as rod–cone dystrophy, impacting both peripheral and central vision. Other ocular symptoms may include night blindness (nyctalopia), blurry vision, and loss of peripheral vision. The extent and pace of retinal disease in BBS vary significantly. Early detection and timely intervention are vital in managing the ocular complications linked to BBS. The clinical presentation of BBS is highly variable, involving multiple organ systems and leading to symptoms such as vision impairment, obesity, polydactyly, kidney abnormalities, learning difficulties, and hormonal imbalances, among others [72,73].

BBS belongs to a group of conditions known as ciliopathies, which arise from dysfunctional cilia, hair-like structures found on the surface of cells. At least 26 genes have been associated with BBS. These genes are responsible for encoding proteins essential for the formation and maintenance of the primary cilium, each contributing to the overall function of the cilia. Mutations in certain BBS genes collectively affect the BBSome, an eight-protein complex consisting of BBS1, BBS2, BBS4, BBS5, BBS7, BBS8/TTC8, BBS9, and BBS18/BBIP1. The movement of molecular cargo within cilia depends on the intraflagellar transport (IFT) system, composed of two stable complexes known as IFT-A and IFT-B. These complexes enable the two-way movement of cargo along the ciliary microtubules. Within the cilium, the BBSome acts as a connector between the IFT machinery and membrane proteins, playing a crucial role in organizing signaling molecules within specific compartments [74]. Disruption of the BBSome causes displacement of membrane receptors within the cilium, leading to improper ciliary transport, ciliopathies, and ultimately, retinal rod–cone dystrophy [75,76,77].

Furthermore, ciliary dysfunction in the outer segment often precedes noticeable retinal changes. ffERG can detect severe abnormalities in rod and cone function even before typical signs of retinitis pigmentosa are visible during a fundus examination [78,79,80,81]. mfERG show the reduction of response densities that starts from the peripheral retina across different stages of BBS [78,82,83].

### 4.2. Alström Syndrome

Alström syndrome is a rare autosomal recessive genetic disorder, first described in 1949. This condition presents with a wide array of clinical symptoms that overlap significantly with Bardet–Biedl syndrome (BBS). To date, the only gene identified as responsible for Alström syndrome is *ALMS1* [84,85,86,87,88,89,90]. The ALMS1 protein is located in centrosomes and basal bodies of ciliated cells, suggesting it plays a role in microtubule organization, particularly in assembling the mitotic spindle, guiding intracellular organelle and vesicle trafficking, and anchoring the axoneme of cilia and flagella. This function in ciliary structure explains the symptom overlap between Alström syndrome and other ciliopathies, such as BBS.

From a clinical standpoint, Alström syndrome is classified as an obesity-related disorder, characterized by features such as infantile-onset dilated cardiomyopathy, progressive sensorineural hearing loss starting after the development of speech, and late-onset diabetes mellitus, typically preceded by hyperinsulinemia. Unlike BBS, Alström syndrome does not involve polydactyly or other limb abnormalities. Patients may also experience renal, hepatic, and pulmonary complications, which often lead to organ fibrosis and contribute to the condition’s poor prognosis. A distinct variant of Alström syndrome has been identified among French-Acadian families in Louisiana, which is also associated with developmental delay, a symptom not commonly seen in other cases of the syndrome.

Ocular involvement is a hallmark of Alström syndrome, with early-onset visual impairment caused primarily by cone–rod dystrophy. Patients typically present in infancy with symptoms such as photophobia, nystagmus, and significantly reduced visual acuity, often accompanied by a bull’s-eye maculopathy. As the disease progresses, the degeneration affects both the cone and rod photoreceptors, leading to further visual deterioration.

In the early stages of Alström syndrome, ffERG reveals significant reduction in cone-driven responses, accounting for early visual symptoms such as decreased central vision, photophobia, and color vision deficits. The reduction in the photopic ERG response is among the earliest indicators of cone dysfunction. As the disease progresses, rod photoreceptor function also deteriorates, as evidenced by the reduced scotopic responses on ffERG. The gradual loss of rod function contributes to night blindness and peripheral vision loss, compounding the visual impairment. However, rod dysfunction typically occurs after significant cone loss, emphasizing the cone–rod pattern of retinal degeneration in Alström syndrome.

Longitudinal ffERG studies demonstrate the progressive nature of retinal degeneration in Alström syndrome, showing a steady decline in both photopic and scotopic responses over time. This allows clinicians to track disease progression and anticipate changes in a patient’s visual function [91,92,93,94,95,96,97,98]. mfERG provides more localized information about retinal function in Alström syndrome, which reveals significantly reduced responses from the central retina early in the disease course. This reduction correlates with the clinical finding of bull’s-eye maculopathy and the decline in visual acuity. The localized data provided by mfERG complements the global data obtained from ffERG, offering a more detailed picture of how the disease affects the central retina.

Complementary imaging modalities, such as OCT and fundus autofluorescence (FAF), provide detailed visualization of retinal layers and photoreceptor integrity, revealing abnormalities like retinal thinning, macular atrophy, or pigmentary changes [98]. Together, these multimodal approaches enable early detection, monitoring of disease progression, and evaluation of therapeutic responses in patients with Alström syndrome.

### 4.3. Cohen Syndrome

Cohen syndrome is a rare autosomal recessive disorder caused by mutations in the *COH1* gene (also known as *VSP13B*) [99,100,101,102]. It is characterized by intellectual disability, distinctive facial features, neutropenia, obesity, and progressive retinal dystrophy. It was first described by Cohen in 1973, and is more prevalent in Finland, the Ashkenazi Jewish population, and the Amish community [99,100,103,104,105,106].

The *COH1* gene is notably large, producing several transcript variants that are differentially expressed across various tissues and have a complex domain structure [107]. Its protein isoforms are believed to play key roles in intracellular vesicle transport and protein sorting, contributing to proper eye development and function. Mutations in *COH1* can disrupt these processes, leading to photoreceptor degeneration and subsequent retinal dystrophy [108]. Duplications are the most common type of *COH1* variants associated with Cohen syndrome [109], presenting a significant diagnostic challenge unless microarray-based techniques or in silico analysis for copy number variants (CNVs) are used during sequencing [110].

**Figure 4:**
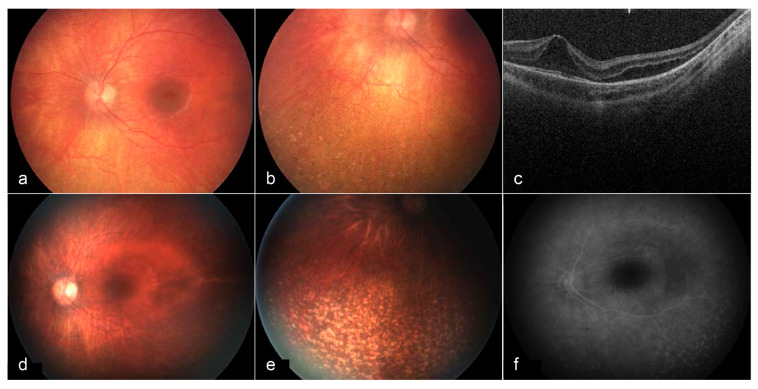
Fundus images and OCT of the left eye at 3 years of age (**a**–**c**) and 6 years of age (**d**–**f**). At age 3, a bullseye maculopathy with surrounding elevation is present. Punched out chorioretinal lesions are present in the periphery (**b**). On OCT, cystoid/schitic changes and disruption of the photoreceptor outer and inner segments are present (**c**). At age 6, macular findings (**d**) and peripheral lesions (**e**) remain stable. Late frame fluorescein angiography shows no leakage at the macula, with prominent staining of peripheral lesions (**f**) [113].

**Figure 5:**
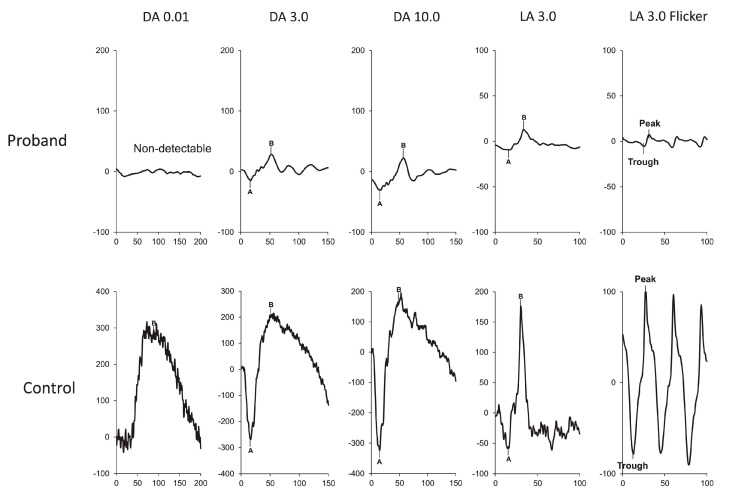
ERG at 3 years of age, showing severe functional reduction in both rods and cones. ERG DA 0.01 response is extinguished. ERG DA 3.0 and DA 10.0 responses are severely decreased. ERG LA 3.0 single flash and 30 Hz flicker responses are severely decreased [113].

From a retinal and visual electrophysiological standpoint, patients with Cohen syndrome typically exhibit a chorioretinal dystrophy similar to retinitis pigmentosa (RP) and early-onset, progressive myopia. Studies on younger patients have shown that retinal abnormalities (Figure 4) and changes in ERG are among the earliest signs of Cohen syndrome. ffERG responses gradually diminish and may become non-recordable in advanced stages (Figure 5) [111,112,113]. This RP-like retinal dystrophy has been reported to be generally absent in Ashkenazi Jewish patients [99,100,111,114]. Also, mfERG could offer additional information about the spatial distribution of retinal dysfunction. A summary of the characteristics of the obesity syndromes and their ocular manifestations are highlighted in Table 4.

## 5. Other Syndromes

### 5.1. Alagille Syndrome (Arteriohepatic Dysplasia)

Alagille syndrome (AGS), also referred to as arteriohepatic dysplasia, is a genetic disorder inherited in an autosomal dominant manner, characterized by high penetrance and variable expressivity. The condition was first described by Alagille et al. in 1975 [115]. It is primarily caused by mutations in the *JAG1* gene [116,117,118,119,120] and, less frequently, in the *NOTCH2* gene [121]. Jagged-1, the protein encoded by *JAG1*, plays a crucial role in the Notch signaling pathway, which is integral to several stages of hematopoiesis and is involved in the regulation and homeostasis of various tissues and organs [122]. *NOTCH2* also functions within this pathway, further demonstrating the interconnected roles of these genes.

The key feature of AGS is chronic liver disease, which often presents in infancy and commonly manifests as neonatal jaundice [123,124]. Other systemic manifestations include distinctive facial features, such as a broad and prominent forehead, pointed chin, bulbous nasal tip, and deep-set eyes. Patients may also exhibit skeletal abnormalities, cardiovascular issues (including cardiac murmurs, pulmonary artery stenosis, and other malformations), renal disease, and additional less common findings. The 20-year survival rate for individuals with AGS is estimated to be 75%, with congenital heart defects being a primary cause of increased mortality up to 10% [125]. There is also a heightened risk for intracranial hemorrhage and liver-related complications.

Ocular manifestations in AGS can include both anterior and posterior segment changes. However, treatment is rarely required, and visual prognosis is usually satisfactory. Common findings include posterior embryotoxon, iris abnormalities, Axenfeld anomaly, Rieger anomaly, optic disc anomalies [71,126,127], diffuse fundus hypopigmentation, and widespread pigmentary retinopathy, in some cases [128,129,130,131,132]. Progressive chorioretinal atrophy [133,134,135], rhegmatogenous retinal detachment [136], focal choroidal excavation [137], optic pit [138], and myelinated retinal nerve fibers [139] in AGS have also been reported. Optic disk drusen and retinal pigment changes have been reported to be found in up to 90% and 32% of patients with AGS, respectively.

From a visual electrophysiology standpoint, patients with AGS who present with retinopathy typically show reduced ffERG rod and cone responses and PERG response [134]. Abnormal VEP can be observed AGS patients with high myopia [140].

### 5.2. Abetalipoproteinemia (Bassen–Kornzweig Syndrome)

Abetalipoproteinemia (ABL), also known as Bassen–Kornzweig syndrome after its initial description by Bassen and Kornzweig in 1950 [141], is a rare autosomal recessive disorder. This condition is primarily defined by an impaired ability to absorb fats and fat-soluble vitamins (particularly vitamins A, D, E, and K) from the diet, which leads to widespread metabolic and neurological complications [142]. ABL is caused by the mutations of the *MTTP* gene, which encodes microsomal triglyceride transfer protein, an essential component for the synthesis and transport of lipoproteins necessary for lipid metabolism [143,144,145].

From a systemic perspective, ABL is characterized by significant fat malabsorption, presenting early in life with symptoms like failure to thrive, chronic diarrhea, and vomiting. As the disease progresses, patients frequently experience spinocerebellar degeneration, resulting in ataxia and other neurological impairments. A hallmark sign of ABL is the presence of acanthocytes—abnormally shaped red blood cells with spiked projections—visible through blood smears. At the biochemical level, patients exhibit strikingly low or even undetectable levels of low-density lipoprotein (LDL) cholesterol, triglycerides, and apolipoprotein B, all of which are crucial for lipid transport and metabolism.

Ocular involvement in ABL presents as a progressive retinal degeneration. The retinopathy observed in ABL patients is clinically and electrophysiologically indistinguishable from non-syndromic RP. In the early stages, ffERG may show significant reductions in the amplitude of rod and cone responses [146,147]. As the disease advances, these responses can become nearly undetectable, reflecting the progressive retinal degeneration typical of this condition. The use of mfERG may also assist in mapping the spatial distribution of retinal dysfunction, though this technique has not been extensively studied in ABL. Since the disease often involves both retinal and central nervous system degeneration, VEP assessments in ABL patients frequently reveal delayed P100 latency, indicating impaired signal transmission along the visual pathway [147]. This finding correlates with the broader neurological deterioration seen in these patients, such as spinocerebellar degeneration, highlighting the systemic nature of the disorder.

Managing ABL involves rigorous systemic monitoring and intervention to prevent the complications associated with fat malabsorption and vitamin deficiencies [145]. Ensuring adequate caloric intake to support normal growth is essential, and patients are typically placed on a low-fat diet supplemented with essential fatty acids. Additionally, high-dose supplementation of fat-soluble vitamins—particularly vitamins A, D, E, and K—is essential for preventing or mitigating the disease’s neurological and gastrointestinal manifestations [145].

From an ophthalmic perspective, vitamins A and E are of particular importance. The vitamin A deficiency seen in ABL contributes to retinal degeneration, and its supplementation is vital for maintaining retinal function and slowing the progression of retinopathy. Vitamin E plays a critical neuroprotective role and has been shown to have significant benefits for the retina. Studies have demonstrated that ABL patients respond particularly well to vitamin E supplementation, with improvements not only in systemic symptoms but also in retinal and optic nerve function [147]. This response is in stark contrast to non-syndromic RP, where treatment options are more limited and typically less effective [148].

Early identification of ABL is important due to its treatable nature, particularly when it comes to preventing severe complications associated with fat-soluble vitamin deficiencies. Visual electrophysiology can play a role in this early diagnosis. Baseline and follow-up ffERG and VEP assessments can detect retinal dysfunction before clinical symptoms become apparent and can be used to track disease progression. Additionally, electrophysiological monitoring allows clinicians to gauge the effectiveness of vitamin supplementation, particularly vitamin E, which has shown considerable promise in preserving retinal function in ABL patients [149,150].

### 5.3. Cockayne Syndrome

Cockayne syndrome (CS) is a rare autosomal recessive disorder, first described by Cockayne in 1936, that is caused by mutations in genes related to excision repair cross-complementing groups, primarily *ERCC6* and *ERCC8* [151,152,153]. The main cellular defect involves an inability to perform transcription-coupled nucleotide excision repair which is responsible for removing UV-induced pyrimidine dimers active genes. The diagnosis can be made by clinical assessment, genetic testing, and laboratory DNA repair tests conducted on fibroblast cultures obtained from skin biopsies.

Based on the clinical presentation of Cockayne syndrome, four main phenotypes have been identified. However, it is now recognized that this disease presents as a highly variable spectrum rather than discrete phenotypes. This syndrome can be characterized by multisystem deterioration, including post-natal growth failure, progressive microcephaly, and developmental delay. Additional clinical features can include sensorineural hearing loss, skin photosensitivity (similar to xeroderma pigmentosum), intellectual disability, dental anomalies, demyelinating neuropathy, and a characteristic appearance referred to as “cachectic dwarfism”. Hypertension and renal disease are common complications that contribute to a reduced life expectancy.

Ocular manifestations of CS include a rod–cone dystrophy-like pigmentary retinal degeneration, initially presenting with a salt-and-pepper appearance and progressing to severe degeneration, optic atrophy (likely secondary), nystagmus, and cataracts (often challenging to treat due to miotic pupils and a high hyperopic, nearly microphthalmic refractive error). Additional ocular features may include xeroderma pigmentosum-like changes in the cornea and eyelids, such as decreased lacrimation and dry eye syndrome, conjunctival hyperemia, corneal opacities, keratitis, band keratopathy, pterygia, and neovascular pannus. Patients are also prone to squamous and basal cell carcinoma of the eyelid (particularly along the margin), eyelid freckling, depigmentation, scarring leading to ectropion and eyelash loss, and melanomas [154,155]. Electrophysiological findings include markedly attenuated FVEP amplitudes, and ffERG responses can range from near normal to non-recordable [156,157]. mfERG show reductions of response densities in the central retina or the whole tested field which varies in different cases [158]. These electrophysiological data can complement imaging modalities to provide a comprehensive understanding of the disease’s impact on the visual system [159].

Currently, only supportive treatments are available for Cockayne syndrome. Regular dermatological and ophthalmological monitoring are important to monitor for UV-induced damage. The prognosis, however, remains generally poor, except for patients with deep intronic genetic variations, who tend to have a better survival outlook [160].

### 5.4. Joubert Syndrome

Joubert syndrome (JS) is a rare autosomal recessive ciliopathy with an estimated incidence of 1 in 100,000. It was first identified in 1969 by pediatric neurologist Marie Joubert [161]. This syndrome shows significant genetic heterogeneity, with more than 30 associated genes. Many of these overlap with those responsible for other ciliopathies, such as Leber congenital amaurosis (LCA), Senior–Løken syndrome (SLS), and Bardet–Biedl syndrome (BBS), all of which share common ciliary dysfunctions. *AIH1*, *INPP5E*, *ARL13B*, and *CC2D2A* are among the genes most frequently associated with Joubert syndrome with ocular involvement [162].

Mutations in these genes result in disrupted ciliary function, leading to retinal degeneration characterized by rod–cone dystrophy. For instance, *ARL13B* encodes a small GTPase localized to cilia. Pathogenic variants in this gene disrupt photoreceptor development and maintenance, leading to progressive vision loss. Similarly, *INPP5E* encodes, a phosphoinositide 5-phosphatase essential for ciliary signaling, undergoes mutations that cause ciliary instability and photoreceptor degeneration [163].

Clinically, JS is characterized by brain abnormalities, most notably the “molar tooth sign” on MRI, as well as ocular and renal involvement. Neurological symptoms may include ataxia, abnormal breathing patterns (such as hyperpnea or sleep apnea), hypotonia, seizures, and abnormal movements of the eyes and tongue. Developmental anomalies like polydactyly, cleft lip or palate, and tongue abnormalities can also occur [164,165].

Ophthalmic involvement in Joubert syndrome often stems from damage to photoreceptor cells, leading to retinal dystrophies that resemble conditions like retinitis pigmentosa (RP) or LCA. Distinguishing the retinal phenotype in JS from related ciliopathies such as LCA, SLS, and BBS, can be challenging. Other ocular manifestations may include chorioretinal colobomas and a Coats-like vascular pathology [162,166,167].

**Figure 6:**
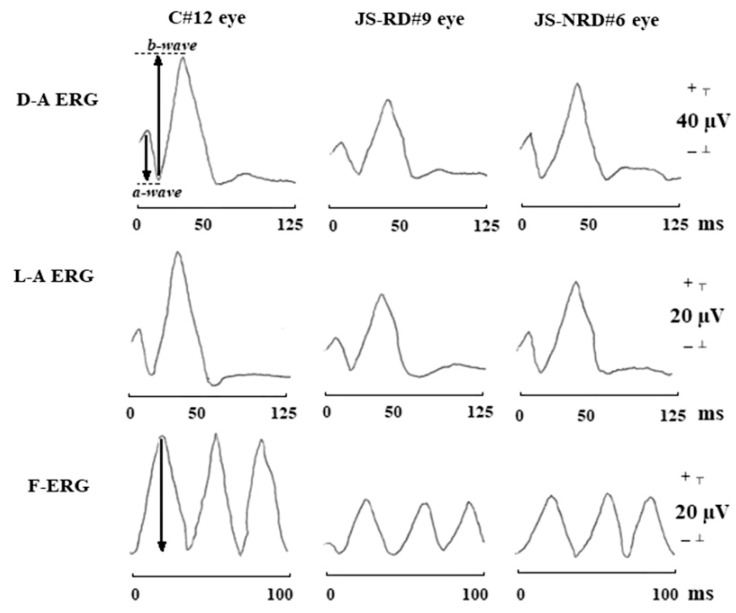
Typical waveforms of dark-adapted ERG (D-A ERG), light-adapted ERG (L-A ERG), and 30-Hz flicker ERG (F-ERG) in a control subject (C#12 eye), in a representative patient with Joubert syndrome with retinal dystrophy (JS-RD#9 eye), and in a representative patient with Joubert syndrome without retinal dystrophy (JS-NRD#6 eye). With respect to control eye, both JS-RD and JS-NRD eyes showed reduction of amplitude and delayed implicit times in D-A ERG, L-A ERG, and F-ERG [171]. The arrows in the D-A ERG waveform show the amplitudes of a-wave and b-wave, respectively. The arrow in the F-ERG waveform shows the amplitude of F-ERG.

In patients with Joubert syndrome, ERG and VEP play a role in assessing the functional integrity of the retina and the visual pathway [168,169,170]. Since photoreceptor cell dysfunction is a hallmark of this syndrome, full-field ERG is often used to evaluate the severity of retinal dystrophy. In those presenting with RP-like or LCA-like symptoms, ERG may show a significant reduction or absence of rod and cone responses (Figure 6), reflecting the degeneration of photoreceptors typical in ciliopathies [171]. When patients exhibit abnormal eye movements or signs of visual pathway involvement, VEP can be used to assess the function of the optic nerve and visual pathways [172]. Abnormal VEP responses in Joubert syndrome patients may reflect delayed conduction or impaired signal transmission, especially in cases with more pronounced neurological involvement.

### 5.5. Mucopolysaccharidosis

Mucopolysaccharidosis (MPS) represents a group of lysosomal storage diseases that primarily affect the central nervous system. Recent advancements in enzyme replacement therapies are showing potential in altering the prognosis and clinical landscape of these diseases [173]. Ocular manifestations are common in several MPS subtypes, many of which exhibit specific retinal abnormalities and distinctive findings on visual electrophysiology.

Mucopolysaccharidosis I (MPS I, Hurler syndrome) is an autosomal recessive condition caused by mutations in the *IDUA* gene, which leads to a deficiency in alpha-L-iduronidase [174,175]. Retinal degeneration is common in this condition and frequently mirrors retinitis pigmentosa with impaired rod-driven responses [175]. ffERG often presents an electronegative pattern, while cone-driven responses may remain relatively intact. A subset of cases demonstrates bull’s eye maculopathy, with marked abnormalities on mfERG reflecting localized retinal dysfunction [176,177]. Retinopathy is found in about 43% of patients, while delayed VEPs are observed in 80% of cases, suggesting possible impairment in the optic pathway [178].

Mucopolysaccharidosis II (MPS II, Hunter syndrome) is an X-linked disorder caused by mutations in the *IDS* gene, which affects iduronate 2-sulphatase [179]. Similar to MPS I, retinopathy in MPS II frequently manifests as RP, characterized by electronegative ffERGs and extensive rod system dysfunction [180]. Approximately 50% of patients exhibit disseminated retinal pigment epitheliopathy [178]. VEPs are delayed in around 67% of patients, indicating delayed optic nerve conduction. There is no evidence of corneal clouding in these cases, making VEPs a more reliable measure of optic pathway involvement.

Mucopolysaccharidosis III (MPS III, Sanfilippo syndrome) is an autosomal recessive condition that is the most common MPS subtype in the United States, accounting for up to 80% of all MPS cases [181]. This disorder is caused by defects in one of four genes, *SGSH*, *NAGLU*, *HGSNAT,* or *GNS,* which are associated with a deficiency in lysosomal enzymes for the degradation of heparan sulfate. Although primarily a neurological condition, ocular involvement is nearly universal in MPS III. Common manifestations include optic nerve swelling and atrophy, alongside broader ocular changes such as corneal clouding, cataract, and pigmented retinopathy with more severe reduction of rod responses than cone responses in ffERG [182].

Mucopolysaccharidosis IV (MPS IV, Morquio syndrome) is another autosomal recessive MPS subtype, associated with pathogenic variants in *GALNS* and *GLB1*. Retinopathy is rarely seen, occurring in only 6% of MPS cases. However, delayed VEPs have been reported in all MPS IV patients [178]. Given the near-universal presence of corneal clouding in MPS IV, it is essential to account for potential confounding effects on VEP interpretation, as delays may reflect optical impairments rather than neural impairments.

Mucopolysaccharidosis VI (MPS VI, Maroteaux–Lamy syndrome) is an additional autosomal recessive form of MPS linked to mutations in the *ARSB* gene, resulting in arylsulfatase B deficiency [183]. Retinopathy is documented in approximately 50% of affected individuals, with delayed VEPs observed in all patients in one cohort study [178]. As with the other MPS subtypes, the presence of corneal clouding complicates the accurate interpretation of VEPs, necessitating careful consideration of corneal contributions when assessing observed electrophysiological findings.

In conclusion, visual electrophysiology, particularly ffERG, mfERG, and VEPs, offers insights into the ocular involvement in various MPS subtypes. Electrophysiological assessments not only for characterizing the extent of retinal and optic pathway dysfunction but also provide valuable information for monitoring disease progression and the effectiveness of emerging therapies. However, it is important to distinguish between retinal and corneal contributions, especially when interpreting VEPs in the presence of corneal clouding.

### 5.6. Neuronal Ceroid Lipofuscinoses (Batten’s Disease)

Neuronal ceroid lipofuscinoses (NCLs), commonly referred to as Batten’s disease, are a group of genetically diverse neurodegenerative disorders. The earliest report of NCL dates back to 1826 by Otto Christian Stengel, with further documentation by Frederick Batten in 1903 [184]. The hallmark of all NCL variants is a genetic defect in lysosomal storage, resulting in the accumulation of autofluorescent lipopigments, such as ceroid lipofuscin, which ultimately leads to neuronal degeneration in affected tissues [185]. It is known to have at least 14 forms with each having a distinct causative gene. These conditions are typically inherited in an autosomal recessive manner, although ceroid lipofuscinosis neuronal 4 (CLN4), a rare adult-onset form of NCL, arises from a dominant mutation in the *DNAJC5* gene [186,187]. Clinically, NCLs manifest with progressive vision loss, cognitive and motor decline, seizures, and premature death. Depending on the age of onset, NCLs are categorized into infantile (INCL), late infantile (LINCL), juvenile (JNCL), and adult (ANCL) forms. Most patients with NCLs become bilaterally blind by the age of 14 and pass away by the age of 30 [188].

Juvenile NCL (JNCL) caused by *CLN3* is the most common subtype. A hallmark of JNCL is severe retinal degeneration affecting both peripheral and central vision, leading to profound visual impairment by the age of 10. Other findings can include optic disk pallor, retinal arteriolar attenuation, and peripheral retinal pigmentation [188]. Patients with JNCL typically experience significant vision loss that is further compounded by systemic complications, such as cardiac conduction abnormalities, which can be fatal without pacemaker intervention. The progression of this disease in the retina is faster than in other tissues [188]. ffERG may show reduced rod and cone responses with electronegative response. PERG show severe reduction of P50 response [189]. mfERG shows reduced responses in the central area [190].

Infantile NCL (INCL) represents the most severe form, with symptoms emerging before the age of 2. Children with INCL suffer from rapid vision loss, psychomotor retardation, and progressive neurodegeneration, often accompanied by microcephaly [191]. The life expectancy for INCL is typically between 6 to 12 years, though some may live into their 40s and beyond. This form is mainly associated with pathogenic variants in the *CLN1* gene, which encodes palmitoyl-protein thioesterase-1 (PPT1). These mutations disrupt lysosomal function and lead to severe cerebral and retinal degeneration [192]. Visual electrophysiology plays a crucial role in the early diagnosis and monitoring of INCL. ffERG often reveals an electronegative pattern due to selective b-wave reduction, indicating post-receptor dysfunction in the early stages. As the disease progresses, there is a profound loss of both a- and b-wave amplitudes in scotopic ERG and photopic ERG, with electronegativity persisting. Alongside histopathological studies, this electrophysiological pattern suggests both pre- and post-synaptic transmission abnormalities between photoreceptors and bipolar cells [193].

**Figure 7:**
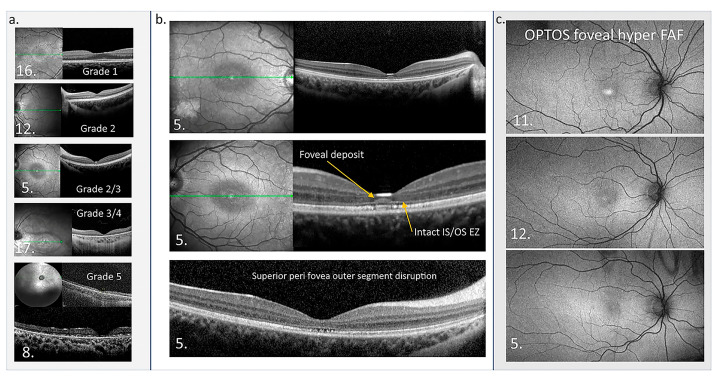
Macular OCT and OPTOS FAF images. (**a**) OCTs of five patients showing the structural disruption with different grades of the maculopathy. (**b**) OCTs from patient 5 in the central panel highlight the atypical accumulation adjacent to the external limiting membrane. The perifoveal disruption of outer segments is also presented. Green line in En Face OCT image: the specific cross-sectional plane within the retinal volume that corresponds to the B-scan image shown on the right. (**c**) OPTOS FAF images are presented from three patients who show foveal hyper FAF while the central thickness is normal at the time of imaging, suggesting foveal hyper FAF is an early sign of retinal dysfunction in CLN2 [195].

**Figure 8:**
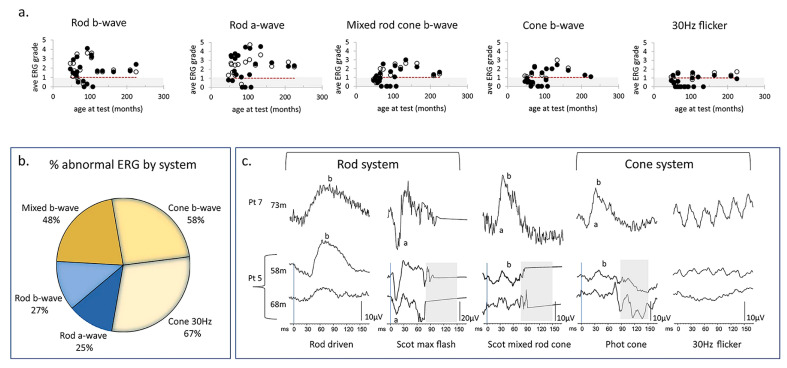
ERG data from children with CLN2 disease. (**a**) Scatter plot shows the changes of the ERG grades calculated by dividing the measured amplitude by the fifth centile reference limit in right eyes and left eyes with age under each of the five ERG conditions. The open and filed circles represent the data from right eyes and left eyes, respectively. Grey region indicates subnormal amplitudes. Red dotted line indicates the trend of the relationship between age and ERG grade. (**b**) Venn diagram shows the larger proportion of abnormal cone ERGs compared to rod ERG. (**c**) ERG waveforms from two patients with CLN2 disease. Pt 7 (hom c.887 G>A) presents the normal ERG amplitudes and waveforms at 73 m. Pt 5 (c.622 C>T, c.1678–1679 del) had subnormal cone ERGs but normal rod ERGs at 58 m. However, there is severe reduction of cone 30 Hz flicker response and moderate decrease of rod responses at 68 m [195].

**Figure 9:**
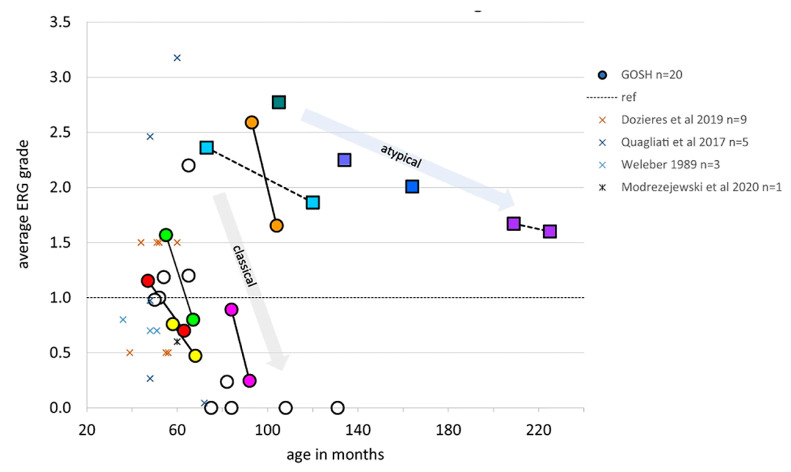
Averaged ERG grades, combined from right eye and left eye for five ERG conditions to provide a single comparable index for each patient, are shown to reduce with age. Dotted line at 1.0 indicates minimum normal range of ERG grade. Circles indicate patients with classical CLN2 disease, boxes indicate those with atypical disease. Serial ERG data from seven patients are linked by lines to show trend of ERG amplitude reduction with age. This grading method allows comparison with other published ERG data obtained by different techniques, which are shown as X symbols [195,197,198,199,200].

**Figure 10:**
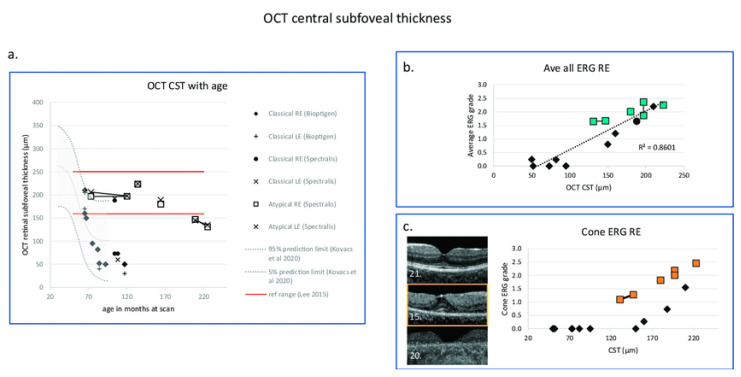
Changes in OCT central subfoveal thickness (CST) and ERG grade with age. (**a**) OCT CSTs are plotted with age and compared with the prediction interval data of right eye redrawn from Kovacs et al. 2020 [201]. GOSH patients with classical disease are indicated by filled symbols, those with atypical disease with boxes. Red horizontal lines indicate ± 3 SD of the CST calculated according to Lee et al., 2015 [202]. (**b**) Right eye OCT central retinal thickness is correlated with average ERG grade from the right eye shown with a linear regression line (Kendall correlation coefficient = 0.71, *p* = 0.003). (**c**) OCT images from three patients with classical CLN2 disease are presented in the (**left panel**). Middle macula image (OCT Bioptigen) in the (**left panel**) (patient 15) has retained the CST, but there is a significant drop out of the ellipsoid zone and reduction of the ERG grade. In the (**right panel**), OCT CST plotted with cone ERG grade shows that those with classical CLN2 disease who retain CST (black-filled symbols) have marked reduction of retinal function compared to those with atypical disease shown by boxes [195]. Black diamond: OCT CST. Green box: Average ERG grade. Orange box: Cone ERG grade. The number at the left lower corner of each OCT B scan is the series number of the patient. GOSH: Great Ormond Street Hospital for Children.

Late infantile NCL (LINCL) is caused by pathogenic variants in the *CLN2* gene, which encodes the enzyme tripeptidyl peptidase 1 (TPP1) [188,194]. Visual symptoms in LINCL typically begin between the ages of 2 and 4, with foveal hyper FAF being an early sign of retinal dysfunction in CLN2 (Figure 7). This rapidly progresses to severe visual impairment, with significant reduction of ffERG rod and cone responses (Figure 8 and Figure 9) [195]. Cone ERGs show functional deficits before CST thinning in classical disease (Figure 10). Without treatment, life expectancy is limited to early adolescence [196]. However, with the advent of cerliponase alfa (Brineura), an enzyme replacement therapy approved in 2017, there is hope for prolonging survival in patients with *CLN2*-related disease. Although Brineura improves neurological outcomes, its effect on visual decline remains uncertain.

Adult-onset NCL (ANCL) is the rarest subtype and typically presents around the age of 30. Its later onset and more variable expressivity result in patients with ANCL experiencing a wide range of disease severity and longevity [196]. ANCL is divided into two types: Type A is associated with mutations in *CLN6* or *PPT1* and predominantly affects the nervous system and ocular system. Patients often exhibit a cone-specific dystrophic pattern on ffERG with preserved rod function alongside a reduction of EOG light peak amplitude that suggests retinal pigment epithelium (RPE) dysfunction [203]. Type B, linked to mutations in the *CTSF* or *DNAJC5* genes, usually has no visual symptoms.

Advances in therapeutic strategies, including gene therapy clinical trials for *CLN2*, *CLN3*, and *CLN6*, provide hope for better treatment outcomes in the future. These developments may also enhance our understanding of the impact of such therapies on the visual manifestations of NCL [196] that can be monitored by visual electrophysiology.

### 5.7. Senior–Løken Syndrome

Senior–Løken Syndrome (SLS) is a rare autosomal recessive genetic disorder, with an estimated prevalence of approximately 1 in 1,000,000 individuals [204]. Two research groups led by Senior and Løken independently described the condition in 1961 [205,206]. SLS is a ciliopathy, arising from defects in the function or structure of cilia, the tiny hair-like structures present on many types of cells. This syndrome primarily affects the kidneys and eyes, though it can have multisystem disruptions.

The most significant feature of SLS is nephronophthisis, a form of kidney disease characterized by the presence of medullary cysts, which often progresses to end-stage kidney failure. Due to the progressive nature of kidney dysfunction, affected individuals may require renal transplantation within the first few decades of life. The retinal symptoms are often similar to those of Leber congenital amaurosis, although not all patients exhibit this presentation. These patients frequently experience photophobia, nystagmus, and hyperopia. The retinal degeneration seen in SLS typically leads to severe visual impairment early in life. This is particularly observed in patients with mutations in genes like *CEP290* (*NHPH6*) and *NPHP5*, which are involved in photoreceptor maintenance [207,208,209,210].

In addition, while the renal and ocular manifestations are hallmarks of SLS, other less common signs may include cone-shaped epiphyses of long bones, sensorineural hearing loss, cerebellar ataxia (impaired coordination), liver fibrosis, and vasopressin-resistant diabetes insipidus.

At least ten genes have been implicated in SLS, all of which are involved in ciliogenesis and the regulation of ciliary protein trafficking [211,212]. These include *NPHP1, INVS, NPHP3*, *NPHP4*, *IQCB1*, *CEP290*, *SDCCAG8*, *WDR19, CEP164*, and *TRAF3IP1* [213,214]. In addition, one study suggests that visual impairment and blindness in Senior–Løken syndrome type 3 associated with the mutations in *Nphp3* might not only result from ciliary dysfunctions but also from malfunctions of the photoreceptor synapse in mice [212].

Ocular involvement in SLS can be quite severe, with conditions such as congenital cataracts and keratoconus (thinning and bulging of the cornea) potentially requiring surgical intervention. The LCA-like retinopathy typically observed in SLS patients can be functionally debilitating, although discrepancies between visual function and retinal structure are observed. This is particularly evident in cases involving *CEP290* mutations. Of note, the *CEP290* gene is also responsible for non-syndromic forms of LCA (specifically LCA10) and some cases of Bardet–Biedl syndrome (BBS14). A summary of the characteristics of the additional syndromes and their changes in electrophysiology are highlighted in Table 5.

## 6. Conclusions

Visual electrophysiology plays a valuable role in diagnosing and monitoring various systemic hereditary syndromes, offering unique insights into retinal and optic nerve function. Electrophysiology’s place in the diagnostic algorithm is pivotal when structural imaging is inconclusive or when functional changes precede visible abnormalities. Electrophysiologic data often complements genetic testing and multimodal imaging, narrowing differential diagnoses and guiding further investigations. As summarized in Table 6, specific electrophysiological findings are associated with particular hereditary conditions, aiding in precise identification and targeted management.

In systemic and local ophthalmic treatments, electrophysiology plays a significant role by quantifying retinal function and monitoring disease progression. In certain conditions, ERG can provide data for assessing therapeutic interventions.

Future prospects for electrophysiology include its integration with advanced imaging modalities and machine learning algorithms for enhanced diagnostic precision. Innovations such as portable ERG devices and non-invasive electrophysiological biomarkers could expand its utility in clinical settings and improve accessibility, potentially transforming the management of hereditary syndromes with ocular manifestations.

## Figures and Tables

**Table 1:** Most commonly used electrophysiology tests.

Test	Purpose	Key Features	Common Applications
ffERG	Assess overall retinal function	Global responses of photoreceptors (a-wave) and bipolar cells (b-wave) in either rod or cone pathways	Detects diffuse retinal disorders
mfERG	Assess localized retinal function	Multiple ERG responses across different retinal regions from photoreceptors and bipolar cells in cone pathway	Identifies localized abnormalities
PERG	Evaluate RGC function	N95 reflects RGC function	Diagnosis of diseases affecting RGC function
VEP	Evaluate function of optic nerve and visual pathway	Measures electrical responses to visual stimuli from visual cortex, reflects the function from ganglion cells to visual cortex	Detects optic neuropathies
EOG	Assess the function of RPE	Measures the standing potential between the cornea and retina related to RPE during dark adaptation and light adaptation	Detects RPE dysfunction

**Table 2:** Characteristics and electrophysiology findings in hearing loss syndromes.

Syndrome	Key Features	Ocular Manifestations *
Refsum Disease	Metabolic disorder (phytanic acid accumulation). Retinal degeneration, hearing loss, ataxia, neuropathy.	Rod–cone RP, ffERG: rod–cone ↓↓, Phytanic acid toxic to rods, secondary cone loss.
USH	AR disorder. Hearing loss, vision impairment (Types I–III). Late-onset ring macular changes in Type IV.	Severe RP. ffERG: rod–cone ↓↓. mfERG: peripheral amplitude ↓↓ and latency ↑.
WS	AR disorder (DIDMOAD): Diabetes insipidus, DM, optic atrophy, deafness. Brainstem atrophy, urinary tract issues.	Rod ERG ↓, Cone ERG ↓↓, VEP latency ↑/amplitude ↓

* ↓: Decrease. ↓↓: Decrease moderately. ↑: Increase.

**Table 3:** Characteristics and electrophysiology findings in mitochondrial diseases.

Syndrome	Key Features	Ocular Manifestations *
MIDD	Diabetes, hearing loss	Spoke–wheel macular RPE changes, mfERG amplitudes in affected area ↓, PERG P50 ↓. FAF, fluorescein angiography are diagnostic.
KSS	Large mtDNA deletions. External ophthalmoplegia, ptosis, systemic issues (heart conduction defects).	Pigmentary retinopathy with “Salt-and pepper” fundus appearance, RPE atrophy. ERG: early rod–cone ↓.
NARP	Night blindness, photophobia, peripheral vision loss, neuropathy, ataxia, hearing loss.	“Salt-and-pepper” fundus appearance, optic atrophy. ffERG: cone–rod responses ↓, non-recordable in advanced stages.

* ↓: Decrease.

**Table 4:** Characteristics and electrophysiology findings in obesity syndromes.

Syndrome	Key Features	Ocular Manifestations *
BBS	Autosomal recessive ciliopathy. Obesity, polydactyly, kidney issues, learning difficulties. Rod–cone dystrophy.	ffERG: rod and cone responses ↓↓↓, mfERG: central ↓.
Alström Syndrome	Autosomal recessive ciliopathy. Obesity, cardiomyopathy, hearing loss, diabetes. Cone–rod dystrophy with photophobia, bull’s-eye maculopathy.	ffERG: cone response early ↓→ rod response ↓, mfERG: start from central retina ↓.
CS	Autosomal recessive ciliopathy-like disorder. Intellectual disability, neutropenia, obesity, RP-like chorioretinal dystrophy. Finnish and Ashkenazi populations.	ffERG: Progressive rod–cone dysfunction

* ↓: Decrease. ↓↓↓: Decrease severely. →: Develop to.

**Table 5:** Characteristics and electrophysiology findings in additional syndromes.

Syndrome	Key Features	Electrophysiology *
AGS	Liver disease, facial anomalies, cardiovascular issues, pigmentary retinopathy, optic disc drusen.	ffERG: ↓, PERG: ↓.
ABL	Fat malabsorption, ataxia, acanthocytosis, RP-like progressive retinal degeneration.	rod/cone ffERG: early ↓→ undetectable, VEP P100 latency: ↑.
CS	Growth failure, microcephaly, photosensitivity, salt-and-pepper retina, cataracts.	Attenuated FVEP, variable ffERG responses.
JS	Ciliopathy with ataxia, breathing abnormalities, brain malformations, RP-like retinal dystrophy.	rod/cone ffERG: ↓, mfERG: ↓, VEP latency: ↑.
MPS	Hurler, Hunter, Sanfilippo, Morquio, Maroteaux–Lamy: RP-like degeneration, optic nerve issues.	ffERG ↓ varies in different subtypes.
NCLs	Batten’s disease, vision loss, retinal degeneration, optic disc pallor, arteriolar attenuation.	rod/cone ffERG ↓, electronegative in INCL, mfERG in JNCL ↓.
SLS	Ciliopathy with kidney disease, LCA-like retinopathy, photophobia, nystagmus.	ffERG ↓↓↓.

* ↓: Decrease. ↓↓↓: Decrease severely. ↑: Increase. →: Develop to.

**Table 6:** Electrophysiology provides findings that indicate specific conditions.

Category	Syndrome	Electrophysiology *	Differentiation
Hearing Loss	USH	Rod ERG ↓↓↓, Cone ERG ↓↓	USH vs. non-syndromic RP
	WS	Rod ERG ↓, Cone ERG ↓↓, VEP latency ↑/amplitude ↓	WS vs. mitochondrial diseases
Mitochondrial	KSS	Rod ERG ↓, Cone ERG ↓	KSS vs. USH/non-syndromic RP
	NARP	Rod ERG ↓↓, Cone ERG ↓↓↓	NARP vs. isolated retinal diseases with systemic features (ataxia, neuropathy)
Obesity	BBS	ffERG: Early/progressive rod and/or cone dysfunction	BBS vs. AS, other ciliopathies
	AS	ffERG: Cone–rod dysfunction	AS vs. BBS
Others	CS	ffERG: Progressive rod–cone dysfunction	CS vs. non-syndromic RP
	MPS	Rod ERG: a-wave ↓↓, b-wave ↓↓↓, Cone ERG intact	MPS vs. syndromic RP
	NCL (BD)	Rod ERG: a-wave ↓↓, b-wave ↓↓, Cone ERG ↓↓↓	Diagnosis with systemic neurodegeneration (seizures, cognitive decline)
	SLS	Rod and Cone ERG ↓↓↓	SLS vs. other retinal degenerations

* ↓: Decrease. ↓↓: Decrease moderately. ↓↓↓: Decrease severely. ↑: Increase.

## Data Availability

Data are contained within the article.
